# Predictors of Severe Sepsis among Patients Hospitalized for Community-Acquired Pneumonia

**DOI:** 10.1371/journal.pone.0145929

**Published:** 2016-01-04

**Authors:** Beatriz Montull, Rosario Menéndez, Antoni Torres, Soledad Reyes, Raúl Méndez, Rafael Zalacaín, Alberto Capelastegui, Olga Rajas, Luis Borderías, Juan Martin-Villasclaras, Salvador Bello, Inmaculada Alfageme, Felipe Rodríguez de Castro, Jordi Rello, Luis Molinos, Juan Ruiz-Manzano, NAC Calidad Group

**Affiliations:** 1 Pneumology Department, ISS/Hospital Universitario y Politecnico La Fe, CIBER Enfermedades Respiratorias (CIBERES), Valencia, Spain; 2 Pneumology Department, Hospital Clínico y Provincial, IDIBAPS, CIBER Enfermedades Respiratorias (CIBERES), Barcelona, Spain; 3 Pneumology Department, Hospital Cruces, Vizcaya, Spain; 4 Pneumology Department, Hospital Galdakao, Vizcaya, Spain; 5 Pneumology Department, Hospital La Princesa, Madrid, Spain; 6 Pneumology Department, Hospital San Jorge, Huesca, Spain; 7 Pneumology Department, Hospital Carlos Haya, Malaga, Spain; 8 Pneumology Department, Hospital Miguel Servet, Zaragoza, Spain; 9 Pneumology Department, Hospital Valme, Sevilla, Spain; 10 Pneumology Department, Hospital Doctor Negrin Las Palmas, Gran Canaria, Spain; 11 Critical Care Department, Hospital Joan XXII of Tarragona and Hospital Vall Hebron, Universtitat Autonoma de Barcelona, Barcelona, Spain; 12 Pneumology Department, Hospital Central Asturias, Oviedo, Asturias, Spain; 13 Pneumology Department, Hospital Germans Trias i Pujol Badalona, Barcelona, Spain; University of Pittsburgh, UNITED STATES

## Abstract

**Background:**

Severe sepsis, may be present on hospital arrival in approximately one-third of patients with community-acquired pneumonia (CAP).

**Objective:**

To determine the host characteristics and micro-organisms associated with severe sepsis in patients hospitalized with CAP.

**Results:**

We performed a prospective multicenter cohort study in 13 Spanish hospital, on 4070 hospitalized CAP patients, 1529 of whom (37.6%) presented with severe sepsis. Severe sepsis CAP was independently associated with older age (>65 years), alcohol abuse (OR, 1.31; 95% CI, 1.07–1.61), chronic obstructive pulmonary disease (COPD) (OR, 1.75; 95% CI, 1.50–2.04) and renal disease (OR, 1.57; 95% CI, 1.21–2.03), whereas prior antibiotic treatment was a protective factor (OR, 0.62; 95% CI, 0.52–0.73). Bacteremia (OR, 1.37; 95% CI, 1.05–1.79), *S pneumoniae* (OR, 1.59; 95% CI, 1.31–1.95) and mixed microbial etiology (OR, 1.65; 95% CI, 1.10–2.49) were associated with severe sepsis CAP.

**Conclusions:**

CAP patients with COPD, renal disease and alcohol abuse, as well as those with CAP due to *S pneumonia* or mixed micro-organisms are more likely to present to the hospital with severe sepsis.

## Introduction

With an incidence of 3–5 cases per 1000 adults/year, Community-acquired pneumonia (CAP), is a frequent cause of death worldwide [1–3]. A complication of CAP is severe sepsis, the syndrome of infection complicated by systemic inflammation and organ dysfunction. Severe sepsis is a worldwide health problem, with an incidence of 343 cases per 100,000 habitants in the USA. At least one-third of CAP patients present to the hospital with severe sepsis.[4,5]

Initial identification of the severity of sepsis is important in order to institute different management and monitoring measures.[6] Clinicians often do not recognize the presence of severe sepsis in CAP patients, even when organ dysfunction is present. Studies aimed at identifying the CAP population at risk of developing severe sepsis in the community before arriving at hospital are lacking.

The aim of our study was to determine the risk factors for presentation at the hospital with severe sepsis in patients with CAP.

## Materials and Methods

### Patients and Data Collection

A prospective, multi-center, observational cohort study was carried out in 13 hospitals belonging to the Spanish national health system (CAP Quality Group); a complete, detailed description has been reported in a prior publication.[7] Briefly, the inclusion criterion was a diagnosis of CAP, defined as acute symptoms or signs with a new compatible radiographic lung infiltrate. Exclusion criteria were nursing-home patients, transplant or oncologic patients, leukopenia or neutropenia (unless attributable to pneumonia), Human Immunodeficiency Virus-positive (HIV) patients with severe immunosuppression (CD4 <100), treatment with corticosteroids (>20 mg/day) or other immunosuppressive drugs, and patients with DNR (do not resuscitate) orders or in whom CAP was considered a terminal event. The study was approved by the ethics committee (ISS Hospital La Fe 2004/15 July, Assent 2004/0101) and the patients provided written informed consent.

We recorded data on age, gender, prior antibiotic treatment for the current episode, comorbid conditions (chronic obstructive pulmonary disease [COPD], heart, liver, neurological or renal diseases, and diabetes mellitus), clinical, analytical and radiological results, and the prognostic scales Pneumonia Severity Index (PSI) [8] and CURB65 risk class. [9]

### Definitions

Comorbidities were assessed based on clinical history along with prior discharge diagnoses and clinical records, review of medications and results of analyses. [8] Sepsis and severe sepsis were evaluated at CAP diagnosis on hospital admission, following previously accepted criteria. [4,7,10] *Sepsis* was defined as the presence of pneumonia and systemic inflammatory response syndrome (SIRS). *Severe sepsis* was considered if criteria for sepsis were met and acute failure of at least one organ was present: arterial hypoxemia (PaO2/FiO2 <300), creatinine >2 mg/dL, acute confusion or hypotension (systolic arterial tension [ST] <90 mmHg). While organ dysfunction has also been defined in terms of hepatic or hematologic failure, information on these organ systems were not available in the data set.

### Microbiological Analysis and Diagnostic criteria

Microbiological studies comprised the following: 2550 (62.7%) blood cultures, 3636 (89.3%*)* urinary antigens for *Legionella pneumophila* and 3654 (89.8%) for *Streptococcus pneumonia*, 1760 (43.2%) sputum cultures, 1902 (46.7%) paired serological studies for *Chlamydophila pneumoniae*, *Mycoplasma pneumoniae*, *Coxiella burnetii* and *Legionella pneumophila*, nasopharyngeal swabs to detect viral nucleic acids, invasive samples obtained by bronchoscopy (285 [7%] bronchial aspirate [BAS] and 118 [2.9%] bronchoalveolar lavage [BAL]), and 276 (6.8%) pleural fluid cultures.

Microbiologic diagnostic criteria were the following: 1) positive urinary antigens for *S pneumoniae* and *Legionella pneumophila;* 2) isolation of microorganisms in BAL (≥10^4^ UFC/mL), BAS (≥10^5^ UFC/mL) or in pleural fluid; 3) isolation of one predominant microorganism in sputum or *L pneumophila* in buffered charcoal yeast extract (BCYE) agar; 4) microorganisms in blood culture; 5) seroconversion or a fourfold antibody increase in titers of IgG for *C pneumoniae* (≥ 1:512), *M pneumoniae* and *C burnetii*, (≥ 1:160) or IgM ≥ 1:32 for *C pneumoniae*, and ≥ 1:80 for *M pneumoniae* and *C burnetii*; 5) positive detection of viral nucleic acids in nasopharyngeal swab.

Mixed etiology was defined as pneumonia due to more than one pathogen (virus or bacteria). [11]

### Outcome Measurements

The evaluated outcome was mortality during hospitalization and at 30-day and 90-day follow-up. Length of stay (LOS) was defined as the number of days from hospital admission to discharge.

### Statistical Study

Data analysis was performed using the SPSS statistical software package, version 15.0. Categorical variable results were expressed as count (percentage) and were compared using the χ^2^ test. Continuous variables were expressed as median with interquartile range (IQR) and were analyzed using non-parametric tests. PSI and CURB65 scales were categorized as low risk (PSI ≤III/ CURB65 ≤2) and high risk (PSI >III/ CURB65 ≥3). Severe sepsis was dichotomized as yes (severe sepsis criteria at hospital admission) and no (non-severe sepsis criteria, the reference group).

Two multivariable statistical studies to predict risk factors for severe-sepsis CAP, the dependent variable, were performed using stepwise logistic regression analyses. In the first model, the included independent variables were those related to characteristics of patients. In the second model, the independent variables were those related to etiology (causal microorganisms). In both models, the independent variables were those found to be significant in the univariate analyses. The Hosmer and Lemeshow goodness-of-fit test was performed to evaluate the adequacy of the models. [12]

## Results

### Study Population

The cohort comprised 4374 patients presenting to the emergency department with CAP and admitted to hospital. We studied 4070 patients after excluding 237 nursing-home and 66 DNR patients: 1529 (37.6%) had severe-sepsis (Table 1).

**Table 1:** Characteristics of CAP with severe sepsis: demographic data, comorbid conditions, radiographic and prognostic scales data.

Characteristics		Severe Sepsis		
		No, n (%)	Yes, n (%)	*p*^c^
Total No.		n = 2,541	n = 1,529	
Demographic data	Age*	69 (50–78)	73 (60–81)	<0.001
	Age ≥65 years	1473 (58.1)	1024 (67.1)	<0.001
	Male gender	1635 (64.3)	1065 (69.7)	0.001
	Current smoker	574 (22.6)	343 (22.5)	0.937
	Alcohol abuse^a^	273 (10.7)	200 (13.1)	0.024
	Prior corticosteroid treatment^b^	95 (3.8)	74 (4.9)	0.083
	Prior antibiotic treatment	651 (25.6)	261 (17.1)	<0.001
Comorbid condition	Diabetes Mellitus	566 (22.3)	294 (19.2)	0.020
	Liver disease	102 (4)	70 (4.6)	0.378
	Heart disease	346 (13.6)	227 (14.8)	0.277
	Renal disease	136 (5.4)	132 (8.6)	<0.001
	Neurological disorders	245 (9.7)	157 (10.3)	0.531
	COPD	494 (19.8)	477 (32.0)	<0.001
Radiographic findings	Multilobar infiltrates	501 (19.7)	427 (27.9)	<0.001
	Pleural Effusion	391 (15.4)	248 (16.3)	0.469
Prognostic scales	PSI (IV-V)	866 (34.1)	971 (63.5)	<0.001
	CURB65 (≥ 3)	531 (20.9)	663 (43.3)	<0.001

Data are presented as number (percentage) unless otherwise indicated.

*Data are presented as median (interquartile range).

^a^ Alcohol abuse: more than 80 g/day.

^b^ Previous corticosteroid treatment: less than 20 mg ∕day prednisone or equivalent.

^c^ *p* value: the χ^2^ test was performed for categorical data and the Mann-Whitney U test was performed for continuous data.

Mortality for the whole cohort was 3.3% and the median length of stay was 7 (IQR 4–10) days. Mortality was significantly higher in severe sepsis CAP (Table 2).

**Table 2:** Length of stay and mortality in CAP with regard to severe sepsis.

		Severe Sepsis				
		No, n (%)	Yes, n (%)	*p* value^b^	OR^c^	95% CI^d^
Total No.		n = 2,541	n = 1,529			
LOS^a^		6 (4–9)	8 (5–12)	<0.001		
Mortality	At 30 days	75 (3)	104 (6.9)	<0.001	2.404	1.773–3.258
	At 90 days	102 (4.2)	127 (8.8)	<0.001	2.194	1.676–2.872

Data are presented as number (percentage) unless otherwise indicated.

^a^ LOS: Length of stay (days). Data are presented as median (interquartile range).

^b^ *p* value: the χ^2^ test was performed for categorical data and the Mann-Whitney U test was performed for continuous data.

^c^ OR: Odds ratio.

^d^ CI: Confidence interval.

### Patient Characteristics

Characteristics related to severe sepsis CAP compared to the reference group are shown in Table 1. Severe-sepsis CAP was more frequent in men, patients older than 65 years, and those with COPD and renal disease, whereas diabetes mellitus was more frequent in those without sepsis. Severe-sepsis CAP also presented with higher PSI and CURB65 scores and more multilobar infiltrates. Patients who received prior antibiotic treatment had lower rates of severe-sepsis.

### Etiology

Etiological diagnosis in the whole cohort was reached in 1506 (37%) patients: 859 (57%) *S pneumoniae*, 104 (6.9%) *L pneumophila*, 44 (2.9%) *C pneumoniae*, 50 (3.3%) *C burnetii*, 50 (3.3%) *M pneumoniae*, 45 (3%) *Pseudomonas aeruginosa*, 43 (2.9%) *Haemophilus influenzae*, 18 (1.2%) viruses, 15 (1%) *E coli* and 121 (8%) mixed etiology.

Severe-sepsis CAP patients had the highest percentage of identified causal microorganisms and more bacteremic episodes. *S pneumoniae* was the most frequent microorganism found, with a higher percentage in severe sepsis. Atypical microorganisms were more frequent in patients with non-severe sepsis, whereas mixed etiology appeared more often in severe-sepsis CAP. Mixed etiology was caused mainly by *S pneumoniae* (29.3% with virus or atypical pathogens, 13.8% with *Pseudomonas aeruginosa* and 5.1% with *S aureus)* (Table 3).

**Table 3:** Etiology of CAP in relation to severe sepsis.

Etiology		Severe Sepsis		
Groups		No, n (%)	Yes, n (%)	*p*^a^
Total No.	Total No. (%)	n = 2,541	n = 1,529	
Known etiology n = 1,507		860 (33.8)	646 (42.2)	<0.001
Gram-positive n = 866		466 (18.3)	400 (26.2)	<0.001
	*S*. *pneumoniae* n = 859 (21.1)	463 (18.2)	396 (25.9)	<0.001
	MRSA n = 7 (0.2)	3 (0.1)	4 (0.3)	0.284
Gram-negative n = 207		123 (4.8)	84 (5.5)	0.358
	*L*. *pneumophila* n = 104 (2.6)	60 (2.4)	44 (2.9)	0.312
	*H*. *influenza* n = 43 (1.1)	25 (1.0)	18 (1.2)	0.559
	*P*. *aeruginosa* n = 45 (1.1)	30 (1.2)	15 (1.0)	0.555
	*E*. *coli* n = 15 (0.4)	8 (0.3)	7 (0.5)	0.466
Atypical pathogens n = 144		102 (4.0)	42 (2.7)	0.034
	*C*. *pneumoniae* n = 44 (1.1)	26 (1)	18 (1.2)	0.645
	*C*. *burnetii* n = 50 (1.2)	37 (1.5)	13 (0.9)	0.089
	*M*. *pneumoniae* n = 50 (1.2)	39 (1.5)	11 (0.7)	0.022
Viruses n = 18		11 (0.4)	7 (0.5)	0.908
Mixed etiology^b^ n = 121		63 (2.5)	58 (3.8)	0.017
Bacteremia n = 284		137 (9.0)	147 (14.5)	<0.001

Data are presented as number (percentage) unless otherwise indicated.

^a^ *p* value: the χ^2^ test was performed for categorical data.

^b^ Mixed etiology is defined as pneumonia due to more than one pathogen (virus or bacteria).

### Multivariable Statistical Analyses

Four independent risk factors related to patients’ characteristics were associated with severe-sepsis CAP: age >65 years, alcohol abuse, renal disease and COPD, whereas prior antibiotic treatment and diabetes were protective factors. With regard to causal microorganisms, *S pneumoniae*, mixed etiology and bacteremia were found to be risk factors (Table 4).

**Table 4:** Multivariable analysis results of severe sepsis related to host factors (first model) and microorganisms (second model).

		Severe Sepsis n = 1,529		
		OR^a^	95% CI^b^	*p*
First model: Host factors				
Demographic data and habits	Age (≥65 years)	1.34	1.15–1.55	<0.001
	Alcohol abuse	1.31	1.07–1.61	0.010
Comorbid condition	Diabetes Mellitus	0.74	0.63–0.88	<0.001
	Renal disease	1.57	1.22–2.03	0.001
	COPD	1.75	1.50–2.04	<0.001
Prior antibiotic treatment		0.62	0.52–0.73	<0.001
Second model: Microorganisms				
Etiology	*S*. *pneumoniae*	1.59	1.31–1.95	<0.001
	*L*. *pneumophila*	1.81	1.14–2.86	0.012
	Mixed etiology	1.65	1.10–2.49	0.017
	Bacteremia	1.37	1.05–1.79	0.021

^a^ OR: Odds ratio.

^b^ CI: Confidence interval.

## Discussion

The most important findings of our study were: 1) 37.6% of hospitalized CAP patients had developed community-onset severe sepsis already at admission; 2) elderly patients, alcohol abusers, patients with renal disease and COPD patients were more likely to develop community-onset severe-sepsis, whereas prior antibiotic treatment was a protective factor; 3) *S pneumoniae* and mixed etiology are the main causal microorganisms of severe sepsis.

Severe-sepsis CAP is not well characterized in terms of the most susceptible population even though it can appear in over one third of the patients. We have identified the aforementioned characteristics, two of them related to comorbid conditions. However, diabetes was more frequent in those without severe sepsis, probably reflecting more lenient hospitalization criteria in diabetic patients.

At hospital admission, patients with severe-sepsis CAP had higher PSI and CURB65 scores, although more than half of these patients had a CURB65 score ≤2, pointing out to the limitations of scales for severity assessment. Patients who had initiated outpatient antibiotic treatment presented a lower frequency of severe-sepsis CAP at hospital arrival. Prior studies have reported the protective effect on mortality when antibiotic therapy was rapidly initiated between 4 and 6 hours after arrival at the hospital. [7,13] Prompt antibiotic administration may rapidly reduce the bacterial load, down-regulating the initial inflammatory cascade and thus decreasing the risk of sepsis. [14,15] On initial severity assessment of CAP, severe sepsis criteria should be taken into account for decision-making process including allocation, monitoring and management. [6]

The multivariable statistical analyses results confirm that alcohol abuse and two comorbid conditions (COPD and renal disease) were independent host risk factors for developing severe-sepsis CAP in the community. The impact of alcohol on developing severe CAP has been linked to an abnormal immune response. [15–18] Curiously, despite the increased risk for severe CAP in COPD patients, mortality is not higher probably due to the use of previous antibiotics and corticosteroids that reduce inflammatory response. [19,20] Our results suggest that patients with alcohol abuse, COPD and renal diseases should be specifically targeted for preventive strategies when in contact with health systems, that is, at discharge or during scheduled outpatient visits. Moreover, if treated as outpatients for CAP, they should be closely monitored and receive instructions to rapidly recognize the signs of sepsis.

Bacteremia and etiological microorganisms are more frequently identified when CAP presents with severe sepsis, most likely due to a higher burden of pathogens in most severe episodes.[2,21] *S pneumoniae* was the most frequently isolated microorganism in severe CAP [1,22,23] and specifically, some serotypes have been independently associated with septic shock.[24] Mixed etiology was the second most common etiology in severe sepsis CAP, underpinning the impact of associated microorganisms on severity. Patients presenting with severe sepsis should benefit of optimizing microbiological tests to rule out bacteremia and mixed etiology, immediately before initiating a combination antibiotic therapy.

This study has some limitations. We have excluded the nursing-home population and patients with CAP considered a terminal event in order to avoid a different population with different characteristics, more frequent nosocomial infections and/or multidrug resistant microorganisms and limited therapeutic efforts; therefore our findings are not applicable to that subset of population. Second, microbiological diagnosis with regard to viruses was incomplete in a considerable subset of patients, the percentage of blood cultures was suboptimal (62.7%), and determination of *S pneumoniae* serotypes was not performed. The indications of microbiological tests in our study relied on the attending physicians. Third, the information regarding septic shock was not recorded. Nevertheless, our strengths are the large sample size and the prospective study design.

## Conclusions

Elderly patients, alcohol abusers and some comorbidities, such as COPD and renal are predisposing conditions for progressing to severe sepsis CAP in the community, mainly due to *S pneumoniae* and mixed etiologies. Those findings may have clinical implications for patients and physicians in primary care and emergency rooms. Preventive CAP strategies such as vaccination–influenza and *S pneumoniae*—and health measures recommended in guidelines should be reinforced in the most susceptible patients. Recognition of severe-sepsis CAP signals should be encouraged for patients and for physicians in primary care and/or emergency rooms. Initial severity CAP assessment could be improved by evaluation of severe sepsis criteria at diagnosis in order to optimize microbiological and analytical tests, to provide closer monitoring and a rapid antibiotic treatment. Efforts should be directed to encouraging actions to reduce the burden of severe-sepsis CAP episodes and facilitate its prompt recognition.
